# Effectiveness of secukinumab in patients with psoriasis and psoriatic arthritis in a Saudi real-world setting

**DOI:** 10.1186/s42358-024-00371-1

**Published:** 2024-04-29

**Authors:** Ibrahim A. Al-Homood, Mohammed Alajlan, Majid Alberdisi, Mohammad Alturki, Ahmed Ali Ahmed, Nancy Zakaria

**Affiliations:** 1 Internal Medicine Department, Medical Specialities Department, King Fahad Medical City, Riyadh, Kingdom of Saudi Arabia; 2 Dermatology Department, King Fahad Medical City, Riyadh, Kingdom of Saudi Arabia; 3 Immunology Medical Affairs, Novartis, Riyadh, Kingdom of Saudi Arabia

**Keywords:** Secukinumab, Psoriasis, Psoriatic arthritis, Real-world, PASI, DAPSA, BSA, DLQI

## Abstract

**Introduction:**

Psoriasis (PsO) is an immune-mediated chronic inflammatory disease that results in severe outcomes that impact the patient’s quality of life and work productivity. We investigated the effectiveness of secukinumab in patients with chronic plaque psoriasis and psoriatic arthritis (PsA) over a 12-month period.

**Methods:**

This was a longitudinal, retrospective study of the medical records of 81 patients with psoriasis and/or psoriatic arthritis who had been treated with secukinumab for at least 12 weeks.

**Results:**

The Psoriasis Area Severity Index (PASI), Body Surface Area (BSA) percentage, and Dermatology Quality of Life Index (DLQI) among patients with PsO and PsO-PsA showed a statistically significant decrease from baseline over 12 months by approximately 9.86, 19.3%, and 9.7, respectively (*p* values < 0.001 for each). Moreover, there was a statistically significant decrease in the overall Disease Activity in Psoriatic Arthritis score (DAPSA) by approximately 22.35 from baseline over 12 months of treatment (*p* < 0.001). Considering the patients who started secukinumab 12 months or more prior to the study cutoff date, the 12-month retention rate was 85%.

**Conclusion:**

In a Saudi real-world setting, secukinumab proved to be an efficient medication with high efficacy and retention rates.

**Supplementary Information:**

The online version contains supplementary material available at 10.1186/s42358-024-00371-1.

## Introduction

Psoriasis (PsO) is an immune-mediated chronic inflammatory disease that affects approximately 0.91% to 8.5% of the world’s population and results in a severe disease burden for psoriasis patients, which impacts their quality of life as well as work productivity [1–3]. Although psoriasis is not contagious, because of its disfiguring and incapacitating symptoms, those who have it frequently struggle socially and psychologically. Additionally, psoriasis is regarded as a serious health issue that needs to be treated properly, as carefully monitored treatment can lower its morbidity and enhance the quality of life.

In 2019, Alshamrani et al. found that the overall prevalence of psoriasis among the study population in the dermatology clinic at King Abdulaziz University Hospital was 5.1% [4]. Another study by Shelleh et al. on patterns of skin diseases in Saudi Arabia stated that psoriasis was present in up to 5.3% of the population in Al-Jouf region [5, 6]. Most patients develop the condition under the age of 30 years, more commonly in men than in women, and it is more prevalent in the northern region of the country [7]. Nevertheless, large-scale epidemiological research on psoriasis symptoms is still uncommon in Saudi Arabia.

Psoriatic arthritis (PsA) is a long-term, systemic inflammatory condition that affects the axial skeleton, peripheral joints, and enthesis and is linked to psoriasis of the skin and nails. Due to the irreversible joint damage and impairment caused by PsA, patients’ everyday functioning and quality of life may suffer [8]. PsA has a reported frequency ranging from 0.3% to 1% in the general population [9, 10], where approximately 30% of people with psoriasis are said to be affected by PsA [11, 12]. A cross-sectional study by Alajmi et al. performed in the western region of Saudi Arabia reported that PsA affected 13.3% of psoriasis patients. According to a review of the disease burden in the Middle East, Saudi Arabia has a 4.3% yearly incidence of PsA among patients with psoriasis [13]. Due to PsA’s variable and diverse clinical presentations, management is extremely challenging.

Existing evidence suggests that IL-17, a proinflammatory cytokine primarily produced by T helper 17 (Th-17) cells, has a role in biological processes that result in inflammation, neutrophilic chemotaxis, and angiogenesis. Keratinocytes, endothelial cells, and innate immune cells are among the main targets of IL-17 in psoriasis. IL-17 causes keratinocytes to express more of the chemokines that draw neutrophils, Th17 cells, and myeloid dendritic cells to the site of psoriatic lesions. Furthermore, IL-17 interacts with endothelial cells to upregulate IL-6, IL-8, and intracellular adhesion molecule-1, which in turn promotes tissue inflammation and procoagulant activity [14]. Meanwhile, IL-17A-producing conventional CD8+ T cells were found to be present in the synovial fluid of inflamed joints in PsA patients, where their levels correlate with disease activity [15]. Based on these investigations, several new biologic medications that specifically target IL-17 have been developed to treat plaque psoriasis and psoriatic arthritis [16]. In the management of psoriatic disease, IL-17 blockade using biologics has yielded remarkable results. Specifically, in the context of psoriasis, it has been demonstrated that IL-17 antagonists have shown higher response rates in achieving Psoriasis Area Severity Index (PASI) scores of 75, 90, and 100 [17]. Additionally, in the case of PsA, IL-17 blockade has been shown to be effective in treating disease manifestations, including nails, skin, peripheral arthritis, dactylitis, enthesitis and axial involvement of psoriatic arthritis [15, 18].

Secukinumab, an established biologic that targets the proinflammatory cytokine IL-17A, was authorized by the US Food and Drug Administration and the European Medicines Agency in 2015 as the first-line systemic therapy for adult patients with moderate-to-severe plaque PsO [19]. It was approved for the treatment of moderate to severe plaque psoriasis and psoriatic arthritis in Saudi Arabia in 2016 and 2017, respectively. Significant clinical trials have demonstrated the sustained effectiveness and safety of secukinumab for up to 5 years in both PsO and PsA. For instance, patients with moderate-to-severe psoriasis showed high and sustained levels of skin clearance and improved quality of life through 5 years in the SCULPTURE trial [20]. Moreover, various clinical trials reported how this medication works to lessen various disease signs and symptoms, such as skin lesions and arthritis, which significantly improves mobility and other quality-of-life measures [21–23].

Comparing secukinumab to etanercept and ustekinumab for the treatment of moderate to severe psoriasis in clinical studies revealed secukinumab to be more effective [24, 25]. Clinical trials have also demonstrated a higher Dermatology Life Quality Index (DLQI) score of 0 or 1 [26, 27] and enhancements in patient-reported symptoms such as itching and pain compared to etanercept and ustekinumab [28]. Moreover, the Future 5 study, a phase 3 study of 150 mg and 300 mg secukinumab with or without loading dose in patients with active PsA, demonstrated sustained clinical efficacy, low radiographic progression and consistent safety over 2 years of treatment, indicating the long-term clinical advantages of secukinumab treatment. This is additionally shown in the trial’s results after 2 years where the resolution of PsA manifestations as enthesitis and dactylitis in patients with symptoms at baseline reached percentages up to 78.0% and 82.8% for the 300 mg group, and 80.3% and 85.5% for the 150 mg group, respectively [29].

Recently, real-world evidence (RWE) has become an essential factor in decisions related to coverage, reimbursement, and formulary inclusions. Notwithstanding the presence of strong positive evidence from randomized controlled trials (RCTs) on the use of secukinumab, it is crucial to show its effectiveness in real-life situations. Since some patients may be excluded from or underrepresented in clinical trials due to substantial comorbidities or prior therapeutic failures, daily practice may frequently differ from how RCTs evaluate the drug’s efficacy and safety. Real-world data on a variety of affected patients are thus required to assess the efficacy and safety of this biologic medication in various populations [23].

Saudi Arabia has a growing population, as there is a common practice of consanguineous marriages, in addition to the difference in Saudi population genetics from the Western population [30]. Therefore, gathering real-world data is crucial to characterize the patterns of secukinumab use in routine clinical practice. Furthermore, generating local RWE will support optimizing patient treatment in addition to efficiency in healthcare delivery. To our knowledge, this is the first study to examine the effectiveness and sustainability of secukinumab in a Saudi real-world setting to analyze its benefit on the Saudi population over one year after the initiation of treatment.

## Methods

### Study design and participants

This was a longitudinal retrospective study to report the patient characteristics at baseline and evaluate the effectiveness and sustainable response of secukinumab in psoriasis and/or psoriatic arthritis patients at their 6- and 12-month follow-up visits who presented to dermatology and rheumatology clinics in King Fahad Medical City Hospital up to December 2022 in a real-world setting. The study population included 81 adult patients, 25 patients with PsO, 7 with PsA, and 49 with both PsO-PsA as per the treating physician. All patients were ≥18 years of age and receiving secukinumab for at least 12 weeks. Patients who were less than 18 years of age, used secukinumab for other indications, or used secukinumab for less than 12 weeks were excluded from this study.

This study was implemented and reported in accordance with the Guidelines for Good Pharmacoepidemiology Practices (GPP) of the International Society for Pharmacoepidemiology (ISPE 2008), the Strengthening the Reporting of Observational Studies in Epidemiology (STROBE) guidelines, and the ethical principles laid down in the Declaration of Helsinki.

The study was approved by the local institutional review board (IRB) at King Abdulaziz City for Science and Technology (KACST) with registration number **H-01-R-012.** Additionally, the IRB registration number with OHRP/NIH, USA is **IRB00010471**, while the approval number Federal Wide Assurance NIH, USA is **FWA00018774**.

### Data collection

Medical charts were reviewed, and data were deidentified and extracted from the hospital’s electronic medical record (EMR) system (“Epic” Software). Data included medical records from the period extending from January 2017 to December 2022.

Outcome measures for PsO included the change in BSA from baseline at enrollment over 6 and 12 months. Other measurements included the DLQI and PASI. Outcome measures for PsA comprised the change in Disease Activity in Psoriatic Arthritis score (DAPSA) from baseline at enrollment to 6 and 12 months.

All study data were accessed in accordance with ethics and research governance requirements. No patient identifiers were collected to ensure the complete protection of patients’ privacy.

### Statistical analysis

Descriptive and analytical statistics were used in this study. After data collection and verification, all data were entered into R statistical software version 4.2.2 “Innocent and Trusting”. Descriptive statistics for baseline patient demographics, clinical characteristics, disease duration, treatment variables, disease severity measures at enrollment, and treatment discontinuation among patients with PSO and/or PsA included the use of mean and standard deviation for normally distributed continuous variables. When a normal distribution was violated, the median and interquartile range were used instead. For categorical variables, counts and percentages were applied. Normality was tested using the Shapiro‒Wilk test.

For the analytical statistics, sensitivity analysis was carried out after dealing with missing data in disease severity measures using the random forest algorithm as a nonparametric approach. It is considered the most appropriate model to impute randomly introduced missing values that appeared in real-world datasets at which several repeated iterations had been performed on the original data to improve the prediction of imputed values until reaching the better-adjusted values.

Aligned rank transformation (ART) ANOVA was used to evaluate the effectiveness of secukinumab on the improvement of outcome measures from baseline at enrollment over 6 and 12 months of treatment followed by pairwise adjustment using the pairwise Wilcoxon test.

For each outcome measure, the results were reported as a total and then subcategorized and analyzed using pairwise comparisons according to the prior use of biologics, whether the patients were biologic naïve or not. For the PsA severity measure, which is the DAPSA score, an additional subcategory was added specifying whether the drug was used in combination with methotrexate (MTX) or alone.

## Results

### Demographics and clinical characteristics of participants

Our study was carried out on 81 patients with PsO and/or PsA who had been treated with secukinumab for at least 12 weeks. Approximately 30.9% of them suffered from isolated PsO, 8.6% suffered from PsA only, and approximately 60.5% had both PSO and PsA (Table 1).

**Table 1:** Descriptive statistics for baseline and follow-up data among patients with psoriasis and/or psoriatic arthritis

Baseline patients’ characteristics		Psoriasis (PsO) N = 25 (30.9%)	Psoriatic arthritis (PsA) N = 7 (8.6%)	PsO-PsA N = 49 (60.5%)	Total N = 81 (100%)
**Demographics**					
Age (years)	Mean (SD)	36.7 (12.9)	41.3 (10.5)	42.7 (13.9)	40.7 (13.5)
Age of diagnosis (years)	Mean (SD)	25.3 (12.4)	30.9 (12.3)	31.8 (14.0)	29.7 (13.5)
Age at starting Secukinumab (years)	Mean (SD)	34.4 (13.2)	39.9 (10.4)	41.3 (13.7)	39.0 (13.6)
Disease duration (years)	Median (IQR)	11.0 (7.0 to 16.0)	4.0 (3.0 to 10.5)	10.0 (6.0 to 13.0)	10.0 (6.0 to 14.0)
Gender	Male	13 (52.0)	3 (42.9)	20 (40.8)	36 (44.4)
Source clinics	Dermatology	25 (100.0)	1 (14.3)	31 (63.3)	57 (70.4)
	Rheumatology	0 (0.0)	6 (85.7)	18 (36.7)	24 (29.6)
**Clinical & comorbidities**					
Diabetes Mellitus		3 (12.0)	1 (14.3)	13 (26.5)	17 (21.0)
Hypertension		2 (8.0)	1 (14.3)	11 (22.4)	14 (17.3)
Ischemic Heart Disease (IHD)		1 (4.0)	0 (0.0)	5 (10.2)	6 (7.4)
Lung diseases		0 (0.0)	0 (0.0)	1 (2.0)	1 (1.2)
Renal disease		0 (0.0)	0 (0.0)	3 (6.1)	3 (3.7)
Depression		7 (28.0)	1 (16.7)	8 (16.3)	16 (20.0)
Uveitis		1 (4.0)	1 (14.3)	0 (0.0)	2 (2.5)
Neurological disease		0 (0.0)	0 (0.0)	2 (4.1)	2 (2.5)
**Treatment variables**					
Previous exposure to biologics		13 (52.0)	3 (42.9)	38 (77.6)	54 (66.7)
Rituximab (RTX)	No	24 (96.0)	7 (100.0)	48 (98.0)	79 (97.5)
	Stopped	1 (4.0)	0 (0.0)	0 (0.0)	1 (1.2)
	Yes	0 (0.0)	0 (0.0)	1 (2.0)	1 (1.2)
Infliximab	Currently	0 (0.0)	0 (0.0)	1 (2.0)	1 (1.2)
	No	25 (100.0)	7 (100.0)	45 (91.8)	77 (95.1)
	Stopped	0 (0.0)	0 (0.0)	3 (6.1)	3 (3.7)
Etanercept	No	22 (88.0)	5 (71.4)	29 (59.2)	56 (69.1)
	Stopped	3 (12.0)	2 (28.6)	20 (40.8)	25 (30.9)
Adalimumab	No	14 (56.0)	5 (71.4)	29 (59.2)	48 (59.3)
	Stopped	11 (44.0)	2 (28.6)	19 (38.8)	32 (39.5)
	Yes	0 (0.0)	0 (0.0)	1 (2.0)	1 (1.2)
Certolizumab Pegol	Currently	0 (0.0)	0 (0.0)	1 (2.0)	1 (1.2)
	No	25 (100.0)	6 (85.7)	46 (93.9)	77 (95.1)
	Stopped	0 (0.0)	1 (14.3)	2 (4.1)	3 (3.7)
Tofacitinib	Currently	0 (0.0)	0 (0.0)	2 (4.1)	2 (2.5)
	No	25 (100.0)	7 (100.0)	45 (91.8)	77 (95.1)
	Stopped	0 (0.0)	0 (0.0)	2 (4.1)	2 (2.5)
Upadacitinib	Currently	0 (0.0)	0 (0.0)	1 (2.0)	1 (1.2)
	No	25 (100.0)	7 (100.0)	48 (98.0)	80 (98.8)
Guselkumab	Currently	4 (16.0)	2 (28.6)	6 (12.2)	12 (14.8)
	No	20 (80.0)	5 (71.4)	41 (83.7)	66 (81.5)
	Stopped	1 (4.0)	0 (0.0)	2 (4.1)	3 (3.7)
Secukinumab	Currently	19 (76.0)	6 (85.7)	37 (75.5)	62 (76.5)
	Stopped	6 (24.0)	1 (14.3)	12 (24.5)	19 (23.5)
Ustekinumab	Currently	1 (4.0)	0 (0.0)	0 (0.0)	1 (1.2)
	No	20 (80.0)	7 (100.0)	42 (85.7)	69 (85.2)
	Stopped	4 (16.0)	0 (0.0)	7 (14.3)	11 (13.6)
Methotrexate	No	15 (62.5)	2 (28.6)	22 (44.9)	39 (48.8)
	Stopped	7 (29.2)	3 (42.9)	18 (36.7)	28 (35.0)
	Yes	2 (8.3)	2 (28.6)	9 (18.4)	13 (16.2)
**Disease severity measures (Baseline)**					
Disease activity at enrollment	Active	13 (52.0)	2 (28.6)	27 (55.1)	42 (51.9)
	Mild	12 (48.0)	5 (71.4)	22 (44.9)	39 (48.1)
Peripheral involvement		0 (0.0)	7 (100.0)	48 (98.0)	55 (96.5)
Axial involvement		0 (0.0)	2 (28.6)	15 (32.6)	17 (31.5)
Swollen Joint Count (SJC)	Median (IQR)	0.0 (0.0 to 0.0)	6.0 (5.0 to 6.5)	6.0 (4.0 to 7.0)	4.0 (0.0 to 7.0)
Total Joint Count (TJC)	Median (IQR)	0.0 (0.0 to 0.0)	9.0 (7.0 to 10.5)	7.0 (5.0 to 9.0)	5.0 (0.0 to 8.0)
Disease Activity in Psoriatic Arthritis score (DAPSA)	Median (IQR)	0.0 (0.0 to 0.0)	29.2 (26.8 to 32.8)	28.1 (25.0 to 33.2)	25.1 (0.0 to 30.2)
Psoriasis Area Severity Index (PASI)	Median (IQR)	18.0 (12.0 to 20.0)	0.0 (0.0 to 0.0)	9.0 (7.0 to 14.0)	11.0 (8.0 to 18.0)
Body Surface Area (%)	Median (IQR)	30.0 (20.0 to 44.0)	0.0 (0.0 to 0.0)	15.0 (8.0 to 30.0)	20.0 (8.0 to 35.0)
Dermatology Quality of Life Index (DLQI)	Mean (SD)	15.4 (4.3)	1.0 (2.4)	10.8 (5.3)	11.5 (6.1)
Human Leukocyte Antigen-B27	Positive	0 (0.0)	0 (0.0)	1 (2.0)	1 (1.2)
	Negative	2 (8.0)	2 (28.6)	6 (12.2)	10 (12.3)
Erythrocyte Sedimentation Rate (ESR)	Median (IQR)	23.0 (16.2 to 52.2)	29.0 (13.0 to 43.0)	36.0 (23.0 to 55.8)	35.5 (22.8 to 53.2)
C-reactive protein (CRP)	Median (IQR)	3.7 (2.3 to 6.6)	1.9 (0.4 to 2.4)	4.1 (1.8 to 7.3)	3.7 (1.8 to 6.7)
**Treatment discontinuation**					
Inflammatory bowel disease secondary to Secukinumab		0 (0.0)	0 (0.0)	2 (4.1)	2 (2.5)
Secukinumab duration (months)	Median (IQR)	21.0 (10.0 to 35.0)	24.0 (10.0 to 25.5)	16.0 (8.0 to 27.0)	18.0 (9.0 to 28.0)
Reason of discontinuation	Non-stop	19 (82.6)	6 (85.7)	37 (77.1)	62 (76.5)
	Active disease	1 (4.3)	0 (0.0)	8 (16.7)	9 (47.4)^a^
	No improvement	2 (8.7)	1 (14.3)	1 (2.1)	4 (21.1)^a^
	Patient controlled	1 (4.3)	0 (0.0)	2 (4.2)	3 (15.8)^a^
3 months follow-up (%)	Median (IQR)	50.0 (40.0 to 60.0)	50.0 (25.0 to 70.0)	60.0 (40.0 to 80.0)	60.0 (40.0 to 70.0)
6 months follow-up (%)	Median (IQR)	70.0 (60.0 to 80.0)	70.0 (60.0 to 87.5)	80.0 (70.0 to 90.0)	80.0 (60.0 to 90.0)
12 months follow-up (%)	Median (IQR)	80.0 (60.0 to 90.0)	75.0 (67.5 to 82.5)	90.0 (80.0 to 100.0)	85.0 (60.0 to 92.5)
18 months follow-up (%)	Median (IQR)	90.0 (75.0 to 100.0)	90.0 (87.5 to 92.5)	90.0 (80.0 to 100.0)	90.0 (80.0 to 100.0)

Data are represented as count (%), mean (standard deviation), or median (interquartile range)

*PsO* Psoriasis, *PsA* Psoriatic Arthritis, *PsO-PsA* Concurrent psoriasis and psoriatic arthritis

^a^Estimated among those stopped Secukinumab (23.5%)

Regarding overall patients’ comorbidity history, approximately 21.0%, 17.3%, 7.4%, and 1.2% had a history of diabetes mellitus (DM), hypertension, ischemic heart disease (IHD), and lung diseases, respectively. Precisely, the PsO-PsA group demonstrated higher comorbid percentages than those with PsO or PsA alone where there was a 26.5% versus 12.0% DM, 22.4% versus 8% hypertension and 10.2% versus 4.0% IHD for PsO-PsA and PsO groups, respectively. However, the depression was higher in the PsO group with 28% vs 16.3% for the PsO-PsA group. More data regarding each group’s clinical characteristics and history of comorbidities can be found in Table 1.

For the baseline measurements, the median PASI score was 18.0 (IQR: 12.0 to 20.0) and 9.0 (IQR: 7.0 to 14.0) for patients with PsO and patients with concomitant PsO and PsA (PsO-PsA), respectively. Additionally, the baseline median BSA percentage for PsO patients was 30.0% with (IQR: 20.0% to 44.0%) and 15.0% with (IQR: 8.0% to 30.0%) for PsO-PsA, while the average DLQI at enrollment for PsO was 15.4 ± 4.3 and for PsO-PsA was 10.8 ± 5.3. For PsO-PsA, the median DAPSA score at enrollment was 28.1 (IQR: 25.0 to 33.2).

Approximately 66.7% of patients were previously exposed to biologics. The median duration of secukinumab treatment among the study population was 12 months (IQR: 9.0 to 34.0). The median patient 6-month follow-up percentage was 80.0% (IQR: 60.0% to 90.0%), and the median 12-month follow-up percentage was 85.0% (IQR: 60.0% to 92.5%). Further details on baseline characteristics, disease severity measures, and patients’ laboratory values can be found in detail in Table 1.

### Effectiveness

No safety concerns have been reported. Each disease severity measure outcome is described in details in the following paragraphs.

#### Body Surface Area (BSA) percentage

PsO and PsO-PsA patients demonstrated a statistically significant decrease in the overall BSA percentage by 17.0% and 19.3% after 6 and 12 months of treatment, respectively, compared to the baseline (*p* < 0.001 for each) (Additional File 1, Fig. 1).

**Fig. 1:**
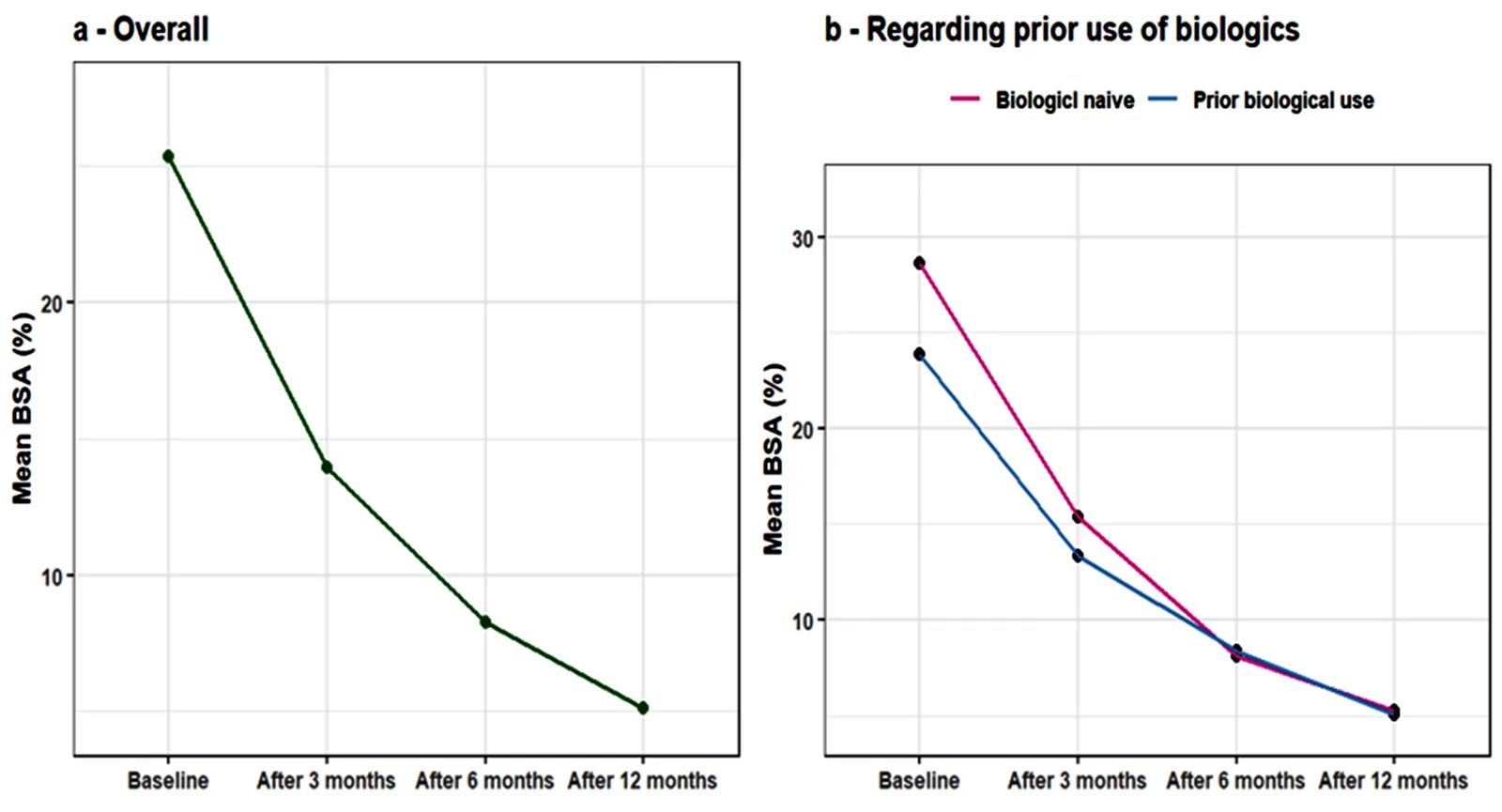
Change in mean Body Surface Area (BSA %) over 12 months among PsO and PsO-PsA patients. **a** Mean BSA improvement. **b** Mean BSA improvement in biological naive patients and patients with prior biological failure

Biologics-naïve patients showed a statistically significant decrease by approximately 22% and 24% after 6 months and 12 months of treatment, respectively, compared to baseline (*p* values < 0.001 for each), while patients who had prior use of biologics showed a statistically significant decrease in BSA percentage by approximately 15% and 17.5% after 6 and 12 months of secukinumab, respectively, compared to baseline (*p* values < 0.001 for each) (Additional File 1, Fig. 1).

#### Psoriasis Area Severity Index (PASI)

The absolute PASI among patients with PsO and PsO-PsA showed a statistically significant decrease using pairwise comparisons by 8.0 and 9.86 after 6 and 12 months of treatment compared to baseline (*p* < 0.001 for each) (Additional File 2, Fig. 2).

**Fig. 2:**
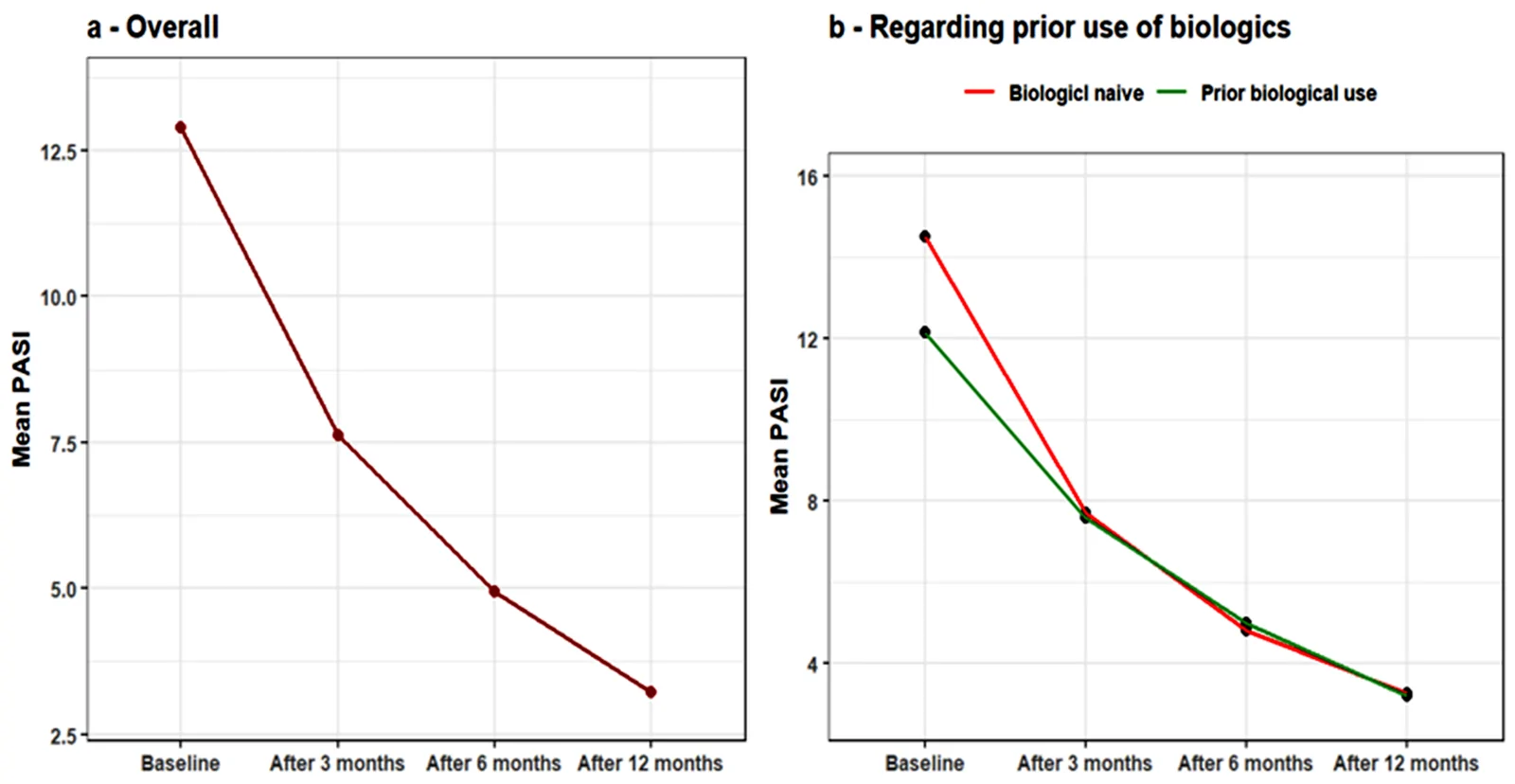
Change in mean Psoriasis Area Severity Index (PASI) over 12 months among PsO and PsO-PsA patients. **a** Mean PASI improvement. **b** Mean PASI improvement in biological naive patients and patients with prior biological failure

Approximately 63.5% and 83.8% of patients reached an absolute PASI ≤ 5 after 6 and 12 months, respectively, while approximately 43.2% and 52.7% of patients reached an absolute PASI ≤ 3 after 6 and 12 months, respectively.

In the case of patients with a prior history of biologics usage, a statistically significant decrease in absolute PASI by approximately 7.5 and 8.8 was observed after 6 and 12 months of secukinumab, respectively, compared to baseline (*p* values < 0.001 for each), while biologics-naïve patients showed a statistically significant decrease in absolute PASI score by approximately 9.09 and 11.6 after 6 months and 12 months of treatment, respectively, compared to baseline (*p* values < 0.001 for each) (Additional File 2, Fig. 2).

#### Dermatology Quality of Life Index (DLQI)

The overall DLQI score in patients with PsO and PsO-PsA showed a statistically significant decrease by 9 and 9.7 after 6 and 12 months of treatment compared to the baseline (*p* < 0.001 for each) (Additional File 3, Fig. 3).

**Fig. 3:**
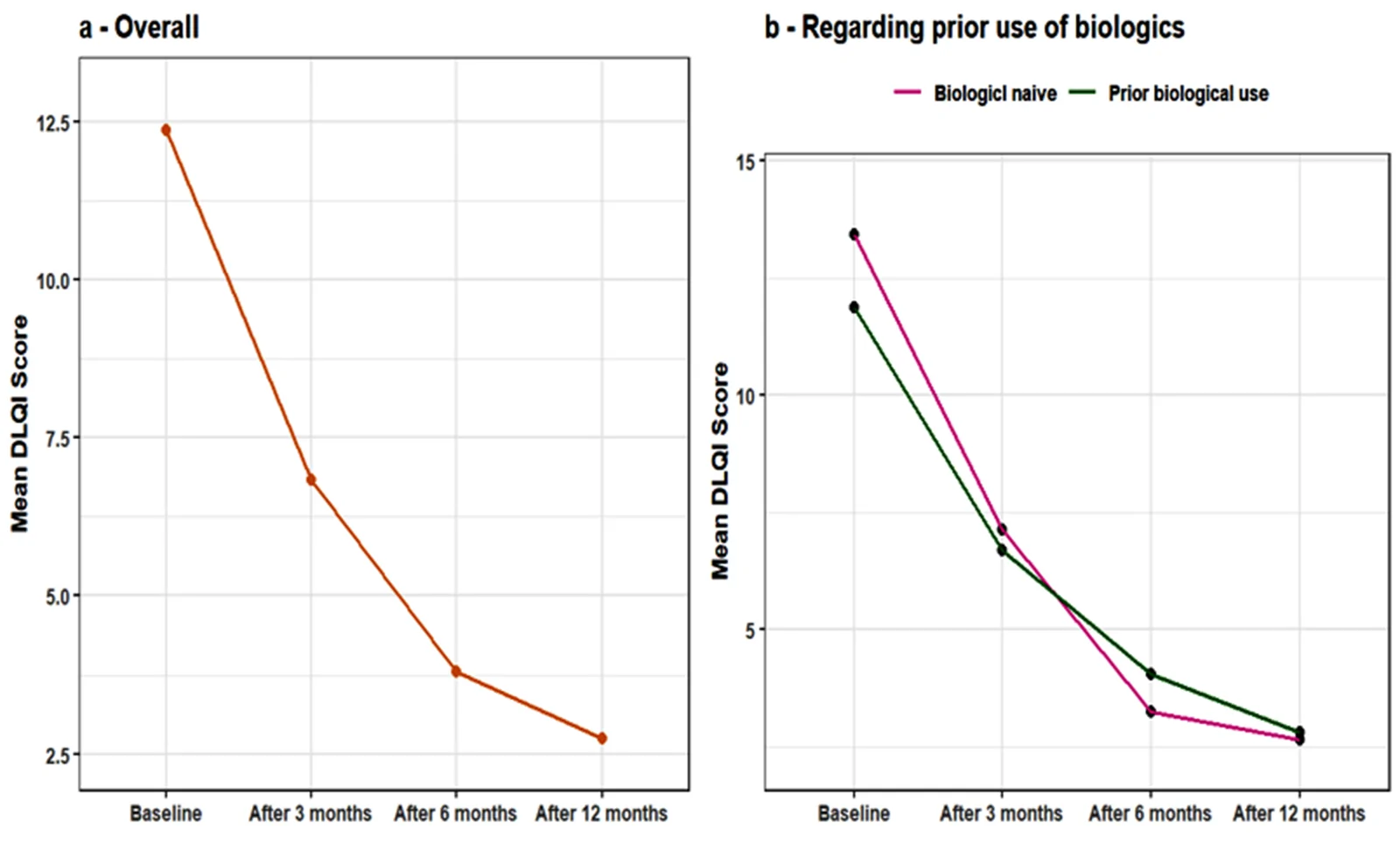
Change in mean Dermatology Quality of Life Index (DLQI) over 12 months among PsO and PsO-PsA patients. **a** Mean DLQI improvement. **b** Mean DLQI improvement in biological naive patients and patients with prior biological failure

After both 6 and 12 months, the overall percentages of PsO and PsO-PsA patients who reached an absolute DLQI of 0 or 1 is 32.4%.

The DLQI score among biologics-naïve patients showed a statistically significant decrease by approximately 10.5 and 10.6 after 6 and 12 months of treatment, respectively, compared to baseline (*p* values < 0.001 for each). Among those who had prior use of biologics, there was a statistically significant decrease by approximately 8.0 and 9.35 after 6 and 12 months of treatment, respectively, compared to baseline (*p* values < 0.001 for each) (Additional File 3, Fig. 3).

#### Disease Activity in Psoriatic Arthritis score (DAPSA)

The overall DAPSA scores among patients with PsA and PsO-PsA patients showed a statistically significant decrease by 21.5 and 22.35 after 6 and 12 months of treatment compared to the baseline (*p* < 0.001 for each) (Additional File 4, Fig. 4).

**Fig. 4:**
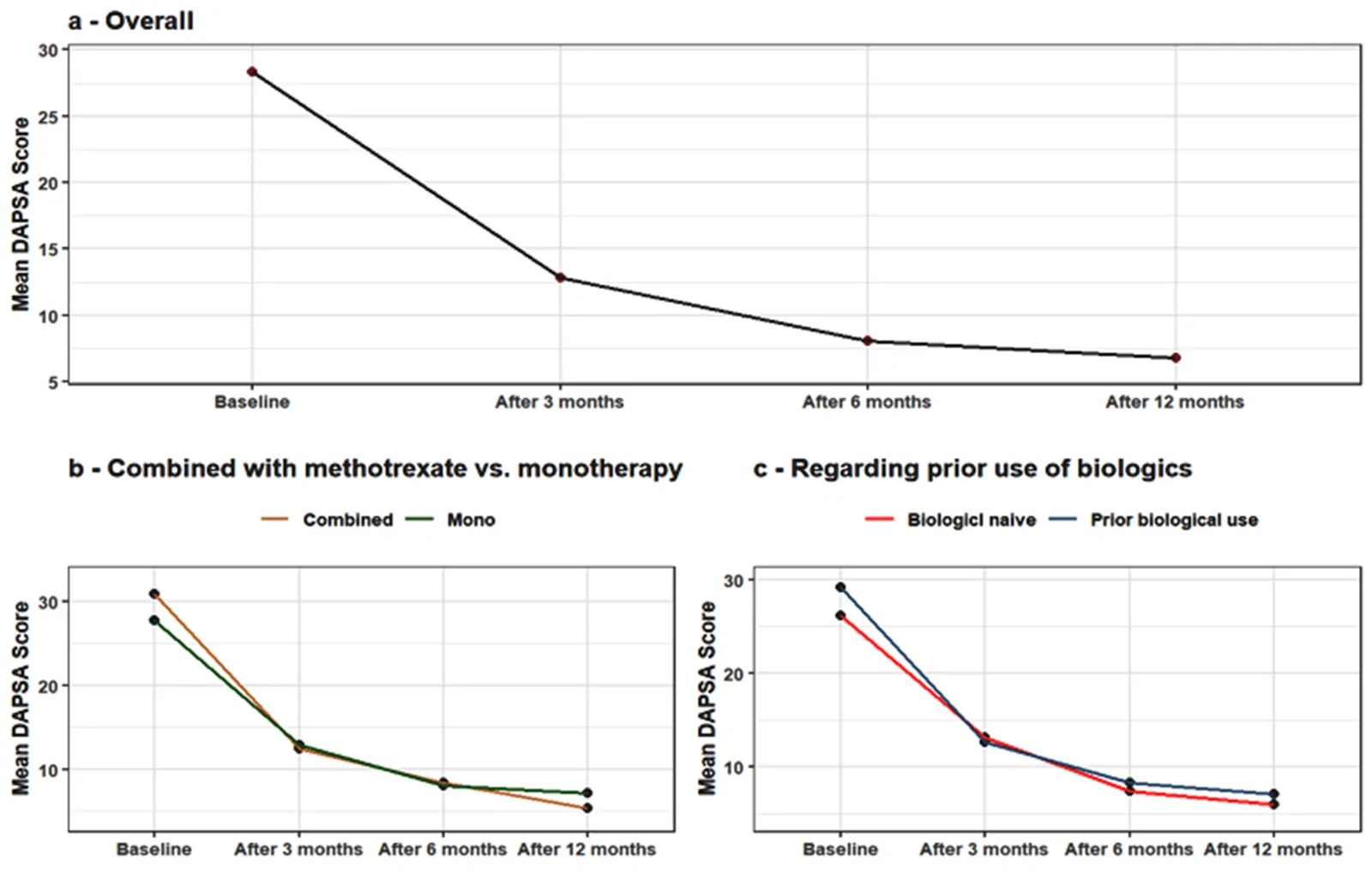
Change in mean Disease Activity in Psoriatic Arthritis score (DAPSA) over 12 months among PsA and PsA-PsO patients. **a** Mean DAPSA improvement. **b** Mean DAPSA improvement with or without MTX. **c** Mean DAPSA improvement in biological naive patients and patients with prior biological failure

The overall percentages of PsA and PsO-PsA patients who reached remission (an absolute DAPSA score ≤ 4) were 17.9% and 32.1% after 6 and 12 months, respectively, while approximately 69.6% and 57.1% of them achieved low disease activity (an absolute DAPSA score from 4 to 14) after 6 and 12 months, respectively.

DAPSA score showed a statistically significant decrease among those who received secukinumab alone by approximately 20.9 and 21.4 (*p* values < 0.001 for each) compared to 23.7 and 25.9 in combination with MTX (*p*-value = 0.012 & 0.006, respectively) after 6 and 12 months of treatment, respectively, compared to the baseline. Moreover, the pairwise comparisons between both groups (monotherapy vs. combination therapy) after 6 and 12 months of treatment showed nonsignificant differences (*p* values = 0.322 & 0.536, respectively) (Additional File 4, Fig. 4).

Biologics-naïve patients showed a statistically significant decrease in the DAPSA score by approximately 19.5 and 20.7 after 6 and 12 months of treatment, respectively, compared to baseline (*p* = 0.001 & < 0.001, respectively), while patients with prior use of biologics showed a statistically significant decrease by approximately 22.2 and 23.0 after 6 and 12 months of secukinumab, respectively, compared to baseline (*p* < 0.001 for each) (Additional File 4, Fig. 4).

### Treatment retention rate

**Fig. 5:**
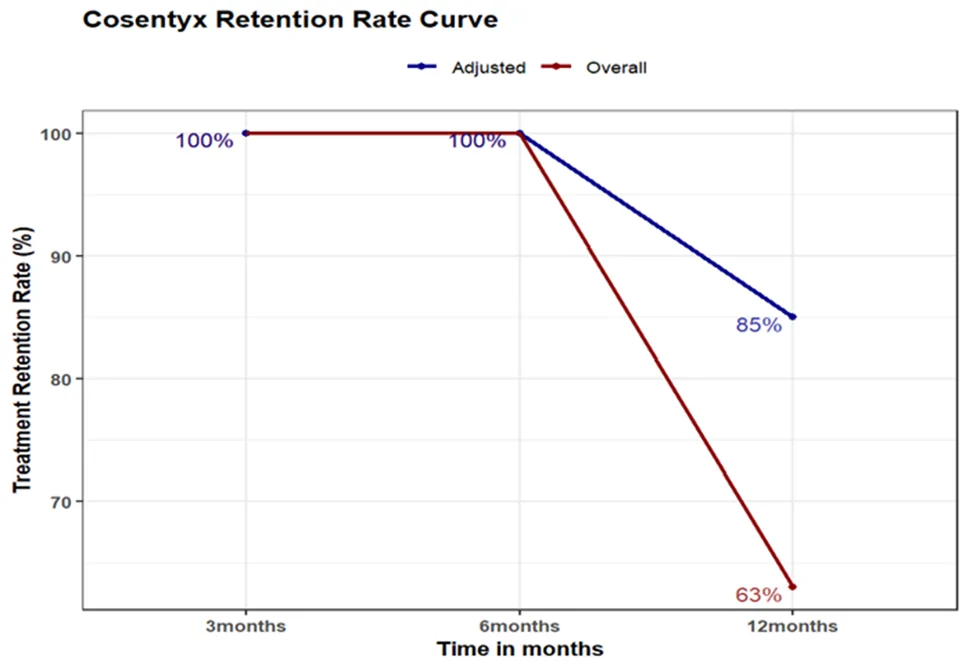
Treatment retention rate curves (overall vs. adjusted for less than 12 months secukinumab)

We determined the “treatment retention rate” to check the percentage of patients who continued to use the drug over 6 and 12 months. The percentage of treatment retention after 6 months was 100%. Considering the patients who started secukinumab 12 months or more prior to the study cutoff date (December 2022), the 12-month retention rate was 85% (Fig. 5).

Approximately 76.5% of the total study population did not stop secukinumab. Considering those 23.5% who did discontinue the treatment, approximately 47.4% of them stopped due to active disease (flare-up), while approximately 21.1% stopped due to lack of improvement. Additionally, 15.8% of these patients stopped treatment as their condition was completely controlled (Table 1).

## Discussion

The introduction of biological drugs has fundamentally revolutionized the treatment of moderate to severe psoriasis during the past few decades due to remarkable progress in our understanding of the pathogenesis of psoriasis [31]. In particular, secukinumab, a fully human monoclonal antibody that specifically neutralizes IL-17A, is highly effective and safe for the treatment of moderate-to-severe plaque psoriasis and PsA [19]. In a real-world setting, patients may be more diverse than those in clinical trials. Our study aimed to determine the effectiveness of secukinumab in PsO and/or PsA patients in a Saudi real-world setting.

The PsO severity measures were also significantly decreased over one year, including the overall PASI, BSA, and DLQI (*p*-value < 0.001 for each). We further evaluated the efficacy of secukinumab in different patient categories to identify the influence of combination therapy or prior biologics use on the treatment outcomes. Outcome measures were analyzed for each group separately to validate our results in each study group and demonstrate all the possible scenarios for secukinumab use.

We found a statistically significant decrease in the overall DAPSA score over 12 months (*p* < 0.001). Additionally, DAPSA decreased significantly in patients regardless of the previous history of exposure to biological agents after 6 and 12 months compared to the baseline (*p* values < 0.001 for each). No safety concerns have been reported.

Galluzzo et al. included 107 psoriasis patients (75% of whom were men) with a mean age of 47.5 years in a retrospective study on the use of secukinumab. The majority of the patients (51.3%) had comorbid conditions, with obesity (23.4%), hypertension (15%), hyperlipidemia (13.1%), and type 2 diabetes (10.3%) being the most prevalent [16]. Similarly, Oguz Topal et al. included 229 patients (60.7% males) and found that the most frequently observed comorbidities were obesity (31%), hypertension (17.9%), hyperlipidemia (12.23%), and diabetes mellitus (11.35%) [32]. Our current study, however, differs in the percentages of comorbidities, with diabetes mellitus being the most common comorbid condition (21%), followed by depression (20%) and hypertension (17.3%).

The history of prior systemic therapy exposure is substantially important. RCTs such as the ERASURE and FIXTURE clinical trials reported that only 12–29% of the patients had previously received biological therapy, and close to two-thirds of the patients did not receive any biological systemic treatment [25]. However, in our current RWE study, approximately 66.7% were previously exposed to biologics, and 16.2% were on methotrexate. These differences further indicate the importance of implementing RWE studies to validate the efficacy of different treatment modalities in real clinical settings versus randomized clinical trials. In a real-world setting, more patients have a history of multiple drug exposures, which gives it considerable significance to test and affirm the optimum treatment strategy in such clinical cases.

Galluzo et al. [33] observed that after 2 years of treatment, only biologics-naïve patients remained significant for PASI score enhancement, and it was the best predictor of outcomes at both 1 and 2 years. Additionally, Ramonda et al. [23] found higher response rates in those not previously treated with biological agents. In contrast, our findings showed a similar decrease in both groups for the PASI score (*p*-value for both biologic-naïve and biologic-experienced groups < 0.001) and the BSA percentage (*p* < 0.001 for each group) after 12 months of treatment compared to baseline. This can be attributed to the difference in ethnicity between the study populations and the patient count in each study group.

Patient-reported outcomes were measured, including the DLQI score, in a study performed by Strober et al. [28], who reported improvements at 6-month and 12-month follow-up visits in both biologic-experienced and biologic-naïve patients. They highlighted that the use of secukinumab as a first-line therapy had favorable outcomes. This is also in agreement with our results, in which both naïve and exposed patients had a significant decrease in the DLQI score at 6 months and 12 months compared to baseline.

As shown by the improvement in BSA percentage or PASI scores in numerous studies, secukinumab has effectively and rapidly improved clinical symptoms while also demonstrating long-term maintenance in the treatment of moderate-to-severe PsO in the real world [16, 34, 35]. This is consistent with our findings where there was a significant decrease in the overall BSA percentage and PASI scores from baseline and over one year, leading to an improvement in the disease condition.

Psoriasis disease measures have been similarly investigated in multiple registries, including the CorEvitas (formerly Corrona) psoriasis registry [36] and the PURE registry [37]. The CorEvitas study described an improvement in all patient-reported outcomes, including BSA and DLQI, after 6 months of treatment. Both biologic-experienced and biologic-naïve patients observed improvements in the DLQI scores by a mean (SD) of 4.3 (6.3) and 5.6 (6.7), respectively. With 12-month follow-up visits, comparable mean (SD) improvements were also seen in patients with biologic experience and biologic inexperience (4.0 [6.1] and 6.6 [6.1], respectively). For the PURE registry, real-world data were collected from patients in Canada and Latin America, where after 12 months, they reported PASI 75, PASI 90, and PASI 100 in 84.4%, 71.1% and 53.3% of patients treated with secukinumab, respectively. Additionally, they noticed an improvement in the DLQI score, where the percentage of patients reaching DLQI 0/1 increased from 6.9% at baseline to 76.5% after 12 months in patients treated with secukinumab [37]. All these observations are in accordance with our study in terms of the efficacy of secukinumab for psoriasis treatment and improvement of dermatology quality-of-life measures.

The EuroSpA registry examined PsA patients from thirteen European countries in a real-life study to determine the effectiveness of secukinumab after 6 and 12 months of use [38]. They utilized the 28-joint Disease Activity Index for Psoriatic Arthritis (DAPSA28) score instead of the regular DAPSA score due to a limited number of patients with the required criteria. Regardless of this limitation, this score was sensitive to change and demonstrated a change in DAPSA28 from baseline to 6 and 12 months by −9.5 and −10.3, respectively (*p* < 0.001 for each). This led to their conclusion of drug efficacy, as it showed a decrease in the PsA measurement scores as well as a high percentage of patients with 6 months of remission. Moreover, they observed overall retention rates of 86% and 76% after 6 and 12 months, respectively [38]. Our current study is aligned with these findings, where our PsA disease measure (DAPSA) showed a significant decrease from baseline by 22.35 over 12 months of treatment. Additionally, we deduced retention rates of 100% and 85% after 6 and 12 months of secukinumab, respectively, further proving the high drug retention rates. Several other studies also noted DAPSA improvement, additionally supporting the efficacy of secukinumab in reducing disease activity in PsA patients [23, 39].

A recently published Canadian real-world study by Gladman et al. [40] demonstrated treatment retention of 73.6% at 12 months. Moreover, an Italian real-life study by Colella et al. [41] showed retention of 75% at the one-year follow-up, and an interim 2-year analysis from SERENA (an RWE study in patients with PsA or ankylosing spondylitis treated with secukinumab) showed a retention rate of 85.2% after one year [42, 43]. This is close to our retention percentage of 85% after a 12-month period, which highlights the fact that the drug retention time after the one-year mark is quite high.

Approximately 16.6% of our study population discontinued secukinumab due to therapy failure, including active disease or no improvement. This rate of discontinuation is significantly less than that noted in other publications, such as the 32-week single-center study conducted by Huang et al. [44] (18.9%), the 52-week retrospective multicenter study conducted by Georgakopoulos et al. [45] (24%), and the 52-week study performed by Notario et al. [46] (21.3%). This can be explained by the difference in study periods or patient count enrolled in each study. However, other studies shared somewhat similar ranges of discontinuation rates as the SCULPTURE trial, which recorded only a 4% discontinuation rate due to inefficacy [20].

### Limitations of the research methods

A potential limitation of this study is the relatively small sample size and short follow-up period. Future studies with larger sample sizes will be helpful to examine the outcomes and validate the current findings. Another common limitation in real-world studies is that the enrolled patients may not be representative of the general population or the whole country of Saudi Arabia, as in our case, they were only recruited from one hospital. Moreover, some data were missing due to the nature of the RWE setup, including the reason for switching to secukinumab, Physician’s Global Assessment (PGA) score for PsO, and Visual Analog Scale (pain-VAS) for PsA.

### Future directions

The generation of Saudi RWE with a large sample size is recommended to further evaluate the efficacy and safety of secukinumab in PsO and PsA patients. Recruitment from multiple hospitals all over the country would be better to yield more generalizable results for the Saudi Arabian population.

## Conclusion

In a Saudi real-world setting, secukinumab proved to be an efficient medication with high efficacy and retention rates up to 52 weeks from starting treatment.

## Electronic supplementary material

Below is the link to the electronic supplementary material.

Additional File 1: Pairwise difference in Body Surface Area (BSA %) from baseline over 12 months among PsO and (PsO-PsA) patients receiving Secukinumab (overall and regarding prior use of biologics)

Additional File 2: Pairwise difference in Psoriasis Area Severity Index (PASI) from baseline over 12 months receiving Secukinumab among PsO and (PsO-PsA) patients (overall and regarding prior use of biologics)

Additional File 3: Pairwise difference in Dermatology Quality of Life Index (DLQI) among PsO and (PsO-PsA) patients from baseline over 12 months receiving Secukinumab (overall and regarding prior use of biologics)

Additional File 4: Pairwise difference in Disease Activity in Psoriatic Arthritis score (DAPSA) among (PsA) and (PsO-PsA) patients from baseline over 12 months receiving Secukinumab (overall, combined with methotrexate or monotherapy, and regarding prior use of biologics)

## Data Availability

The datasets generated and/or analyzed during the current study are not publicly available but are available from the corresponding author on reasonable request.

## List of abbreviations

PsO: Psoriasis
PsA: Psoriatic arthritis
PsO-PsA: Concomitant psoriasis and psoriatic arthritis
BSA: Body Surface Area
PASI: Psoriasis Area Severity Index
DLQI: Dermatology Quality of Life Index
DAPSA: Disease Activity in Psoriatic Arthritis score
RWE: Real-world evidence
RCTS: Randomized controlled trials
GPP: Good Pharmacoepidemiology Practices
ISPE: International Society for Pharmacoepidemiology
STROBE: Strengthening the Reporting of Observational Studies in Epidemiology
EMR: Electronic medical record
ART: Aligned rank transformation
DM: Diabetes mellitus
IHD: Ischemic heart disease
